# RNAi screen reveals synthetic lethality between cyclin G-associated kinase and FBXW7 by inducing aberrant mitoses

**DOI:** 10.1038/bjc.2017.277

**Published:** 2017-08-22

**Authors:** Saoirse O Dolly, Mark D Gurden, Konstantinos Drosopoulos, Paul Clarke, Johann de Bono, Stan Kaye, Paul Workman, Spiros Linardopoulos

**Affiliations:** 1 Cancer Research UK Cancer Therapeutics Unit, Division of Cancer Therapeutics, The Institute of Cancer Research, London, UK; 2 Royal Marsden Hospital, London, UK; 3 Breast Cancer Now, Division of Breast Cancer Research, The Institute of Cancer Research, London, UK

**Keywords:** FBXW7/FBX7, cyclin G-associated kinase, kinome, RNAi, screen aberrant mitoses

## Abstract

**Background:**

F-box and WD40 repeat domain-containing 7 (FBXW7) is an E3 ubiquitin ligase involved in the ubiquitination and degradation of multiple oncogenic substrates. The tumour suppressor function is frequently lost in multiple cancers through genetic deletion and mutations in a broad range of tumours. Loss of FBXW7 functionality results in the stabilisation of multiple major oncoproteins, culminating in increased cellular proliferation and pro-survival pathways, cell cycle deregulation, chromosomal instability and altered metabolism. Currently, there is no therapy to specifically target FBXW7-deficient tumours.

**Methods:**

We performed a siRNA kinome screen to identify synthetically lethal hits to FBXW7 deficiency.

**Results:**

We identified and validated cyclin G-associated kinase (GAK) as a potential new therapeutic target. Combined loss of FBXW7 and GAK caused cell cycle defects, formation of multipolar mitoses and the induction of apoptosis. The synthetic lethal mechanism appears to be independent of clathrin-mediated receptor endocytosis function of GAK.

**Conclusions:**

These data suggest a putative therapeutic strategy for a large number of different types of human cancers with FBXW7 loss, many of which have a paucity of molecular abnormalities and treatment options.

F-box and WD repeat domain-containing 7 (FBXW7) is one of the most frequently perturbed proteins in the ubiquitin–proteasome system in cancer. The FBXW7 is an E3 ubiquitin ligase that degrades an extensive list of oncogenes (Welcker and Clurman, 2008; Inuzuka *et al*, 2011; Wertz *et al*, 2011). It acts as a bridging molecule by binding substrates, through its WD40 domain, and scaffold proteins through its F-box domain. This produces the SCF^FBXW7^ complex that interacts with the ubiquitin-conjugating complex facilitating the transfer of ubiquitin onto the substrate signalling its degradation by the 26S proteasome.

The SCF^FBXW7^ complex is responsible for the degradation of multiple oncogenic substrates including: cyclin E, c-MYC, c-JUN, NOTCH and MCL-1, as well as a plethora of other proteins (Davis *et al*, 2014). The FBXW7 controls the cellular levels of multiple proteins involved in cell cycle regulation, cell proliferation, differentiation, signal transduction, lipid metabolism and anti-apoptosis. Loss of FBXW7 function results in reduced substrate degradation and their overexpression, culminating in cell proliferation, cell cycle deregulation, chromosomal instability and activation of pro-survival pathways, as well as being associated with poor clinical outcomes and drug resistance (Welcker and Clurman, 2008; Wertz *et al*, 2011; Davis *et al*, 2014).

The FBXW7 gene locus 4q32 is deleted in a third of cancers (Knuutila *et al*, 1999) and copy loss occurs in 15% (Kan *et al*, 2010). Multiple studies have detected FBXW7 somatic mutations in a wide range of tumours, including ∼40% of cholangiocarcinomas and T-cell acute lymphoblastic leukaemias, as well as in 4–10% of carcinomas of the endometrium, colon, breast and stomach (Spruck *et al*, 2002; Akhoondi *et al*, 2007). Targeting tumour cells with loss of the FBXW7 presents an important treatment strategy given its widespread involvement in carcinogenesis. Due to the inherent complexities of attempting to restore protein function, synthetic lethality provides a feasible strategy to identify vital proteins that cells have become dependent on, in the absence of the FBXW7 tumour suppressor, that can be inhibited to cause selective cancer cell death. This approach has been clinically validated through the identification of PARP inhibitors for BRCA-deficient tumours (Farmer *et al*, 2005; Ashworth, 2008).

Herein, we describe a high-throughput RNA interference (RNAi) kinome screen using HCT116 FBXW7-deficient cells that contain the targeted deletion of the F-box domain and their wild-type comparators (Jallepalli *et al*, 2001; Rajagopalan *et al*, 2004). We identify cyclin G-associated kinase (GAK) as a potential synthetic lethal partner with FBXW7 deficiency. The identification of the druggable target GAK capable of selective toxicity in FBXW7-deficient cancers has broad therapeutic applications across numerous tumour types.

## Materials and methods

### Cell lines and compounds

Cell lines obtained from ATCC (Teddington, UK) were maintained according to the supplier’s instructions. Authenticated isogenic FBXW7 HCT116 human colon cancer cells were a gift from Professor B Vogelstein (Johns Hopkins Medical Institution, Baltimore, MD, USA). Compound Pitstop 2 (Abcam, Cambridge, MA, USA), Annexin V (BD Biosciences, Franklin Lakes, NJ, USA), propidiuim iodide (Sigma, St Louis, MO, USA) and DAPI (Invitrogen, Waltham, MA, USA).

### siRNA

All siRNAs were used at 50 nmol final concentration (Dharmacon, Lafayette, CO, USA). Primary screen used the siGENOME SMARTpool human protein kinase library arrayed into 96-well plates. All deconvolution and subsequent RNAi were undertaken using the Dharmacon catalogue. The individual GAK siRNAs numbered 1–4 refer to siGENOME (SIG) and 5–8 ON-TARGET plus (OTP). Control siRNAs included ON-TARGET plus nontargeting (NT) and death (siTOX) control.

### Antibodies

The following antibodies were used: *α*-tubulin (Sigma), aurora A (Cell Signaling, Danvers, MA, USA), cleaved PARP (Cell Signaling), clathrin heavy chain (Abcam), FITC-conjugated mouse (Sigma) secondary antibody, GAPDH (VWR International, Radnor, PA, USA), GAK mouse monoclonal (R&D Systems, Minneapolis, MN, USA), HRP-conjugated secondary antibodies (Sigma) and MPM-2 (Millipore, Billerica, MA, USA).

### RNAi for kinome screen and deconvolution

Cells at low passage were split the day before reverse transfection. Conditions were optimised for each cell line so the NT controls were ∼90% confluent by inspection under the microscope and the siTOX was <10% relative to the NT controls on the day of measurement. Screen conditions were as follows: siRNA was mixed with transfection mixture of 0.19% Dharmafect 2 (Dharmacon) in OptiMEM (Invitrogen), aliquoted into 96-well plates in duplicate for two comparator cell lines. After 25 min of incubation at room temperature, 3000 FBXW7^+/+^ and ^−/−^ HCT116 cells in antibiotic-free medium were added respectively and supplemented with double the volume of medium with antibiotics at 7 h. On day 5, medium was removed and 100 *μ*l of CellTiter Glo (Promega, Madison, WI, USA) added, incubated at room temperature before quantifying luminescence using a Victor X5 Plate Reader (PerkinElmer, Waltham, MA, USA). Conditions were scaled up for 6-well plates using 50 nmol of siRNA, 0.25% Dharmafect 2 for 400 000 HCT116 cells.

### Immunoblot analysis

Whole-cells extracts were prepared in Triton lysis buffer (120 mmol NaCl, 50 mmol Tris pH 7.4, 1% Triton, 50 mmol NaF, 1 mmol EDTA, protease inhibitors). Protein concentration of supernatent was quantified using the Bio-Rad protein assay (Hercules, CA, USA). Equal amounts of protein (30 *μ*g) were loaded onto precast 4–12% Bis-Tris gels (Invitrogen) with a rainbow molecular weight marker (GE Healthcare, Little Chalfont, UK) as a size reference and separated by SDS–polyacrylamide gel electrophoresis. Proteins were transferred to nitrocellulose membranes (Sigma), blocked and probed with primary antibody diluted 1 in 1000 in TBS-Tween with 4% skimmed milk overnight at 4 °C. Secondary antibiodies were diluted 1 in 5000 in TBS-T with 4% skimmed milk and incubated for 1 h at room temperature. Protein bands were visualised using ECL chemi-luminescence reagents followed by exposure on Kodak BioMAX film (Rochester, NY, USA).

### Validation of RNAi gene silencing and viability assays

Validation of RNAi gene silencing was determined by immunoblot analysis and viability assays using individual oligonucleotides using 6- and 96-well plate siRNA conditions, respectively. Long-term cell survival assay was as follows: following forward siRNA transfection, 1000 cells were seeded in 6-well plates, complete media were replaced the next day and thrice weekly until day 14 when surviving cells were washed with PBS, fixed with 10% trichloroacetic acid (TCA) for 2 h, washed with water and stained with sulforhodamine B (SRB) for a minimum of 30 min. Wells were washed with 1% acetic acid and dried overnight before taking images. The SRB was eluted using 1 ml of 10 mmol Tris pH 10.5 per well, plates shaken for 5 min before transferring 100 *μ*l of dye to a 96-well plate and absorbance read at 490 nm by the Victor X5 Plate Reader (PerkinElmer).

### Cell cycle profiling by flow cytometry

All cells were harvested from 10 cm dishes using PBS and then trypsin, centrifuged at 1000 r.p.m. for 5 min, supernatant removed and the cell pellet resuspended in 150 *μ*l of PBS to form a single-cell suspension. Cells were fixed using ethanol (−20 °C) to a final concentration of 75% while gently vortexing and stored at −20 °C for 16 h minimum before analysis. Thereafter, cells were washed with PBS, centrifuged at 1000 r.p.m. for 5 min and the supernatant removed. Cell pellets were resuspended in MPM-2 antibody solution and incubated at 4 °C for 1 h, washed with PBS, centrifuged as before and resuspended in FITC-conjugated secondary antibody (1 : 1000 in PBS) and incubated in the dark at 4 °C for 1 h. Cells were washed in PBS, centrifuged and resuspended in 500 *μ*l propidium iodide (PI) solution: 40 *μ*g ml^−1^ PI, 50 *μ*g ml^−1^ RNase A in PBS (Sigma). After 30 min of incubation at room temperature, cells were analysed using the FACS analyser (BD Biosciences).

### Annexin V FITC assay

Reverse transfection using NT control and GAK siGENOME SMARTpool siRNA was undertaken in 6-well plates using FBXW7^+/+^ and ^−/−^ HCT116 cells. Collected cells were ethanol fixed as per cell cycle protocol. Cell pellets were resuspended in 500 *μ*l medium containing 0.2% PI, 0.5% Annexin V and 0.25% calcium chloride. After 15 min of incubation in the dark at room temperature, cells were transferred for FACS analysis (BD Biosciences).

### Immunofluorescence microscopy

Cells were seeded onto 19 mm poly-L-lysine-coated coverslips and cultured for 24 h before being washed with PBS and fixed in either 4% formaldehyde in PEM buffer for 10 min at room temperature or 100% methanol for 10 min at −20 °C. Coverslips were washed in PBS containing 0.1% Triton X-100 (PBS-T) and incubated with glycine blocking solution for 5 min at room temperature. Primary antibodies, diluted in blocking solution, were placed onto each coverslip and incubated at room temperature for 1 h. Followed by washing in PBS-T and incubation with secondary antibodies for 30 min in the dark, repeat PBS-T wash and incubation in DAPI for 3 min, both at room temperature. Following final PBS-T wash, cells were mounted onto a glass slide, edges sealed and viewed using a fluorescence microscope (Zeiss LSM 710, Oberkochen, Germany). Where needed, *z*-stacks were performed comprising 10–30 images at 0.1–0.5 *μ*m intervals, image were captured and deconvoluted image stacks were projected on a single plane using Volocity software (PerkinElmer).

### Live-cell time-lapse imaging

For generation of stable histone H2b-mCherry HCT116 cells, 6 × 10^5^ HCT116 cells were seeded in a 6-well plate day before forward transfection. The medium was aspirated and replaced with 500 *μ*l optiMEM and transfection mixture was added (2 *μ*g of mCherry plasmid with 5 *μ*l of L2000 transfection reagent in 500 *μ*l of optiMEM) and incubated at room temperature for 30 min. At 5 h, the medium was replaced with full medium containing antibiotics. The cells were expanded and then harvested and the mCherry-positive cells were selected using flow cytometric sorting and repeated twice to ensure a high percentage of expressing cells were isolated. For time-lapse imaging, cells were cultured in 96-well Ibidi plates (Martinsried, Germany). Images were taken with a Nikon (Tokyo, Japan) Eclipse TE2000-5 microscope, during which cells were maintained at 37 °C in a humidified stream of 5% CO_2_. Images were taken every 3 min for 24 h with a 40 × objective. The FITC fluorescence was used for histone H2b-mCherry cells, with anaphase onset judged as the first frame in which the sister chromatids moved to the opposite spindle poles. Time-lapse images were analysed using ImageJ software (Bethesda, MD, USA) counting the number of frames from nuclear envelope break down to anaphase onset and data analysed by GraphPad Prism (La Jolla, CA, USA).

### Cell viability assays for Pitstop 2 treatment

A total of 2000 cells were seeded in 100 *μ*l of medium in 96-well plates and incubated overnight at 37 °C. Two-fold serial dilutions of Pitstop 2 (Abcam) in medium were prepared with a DMSO control. An aliquot (50 *μ*l) of drug or DMSO preparation was added to the cells and incubated at 37 °C for 4 days when cell viability was quantified using the CellTiter-Glo assay, as described. Survival fractions were calculated by dividing the raw luminescence values for the drug treated well by the DMSO controls. Data were analysed using GraphPad Prism and survival curves determined.

### Statistical analysis

#### siRNA high-throughput screen statistical analysis

Raw luminescence readings for each well were log transformed and normalised to the NT controls on that plate. The effect of the SMARTpool targeting each gene on cell viability was expressed as a Δ*Z*-score. First the *Z*-score was calculated, estimating the s.d. of the normal distribution of the screen from the median absolute deviation of all 720 SMARTpools, adjusted by a factor of 1.4826 (Abdi, 2007). The Δ*Z*-score was calculated by subtracting the *Z*-score for the FBXW7 wild-type from the deficient HCT116 cells. The mean Δ*Z*-score for the two runs was calculated for each SMARTpool. The *Z*′ factors were obtained using the NT and siTOX control wells, as a marker of the discriminatory power of the screen.

## Results

### RNAi kinome screen identifies GAK as a putative synthetic lethal partner with FBXW7

**Figure 1:**
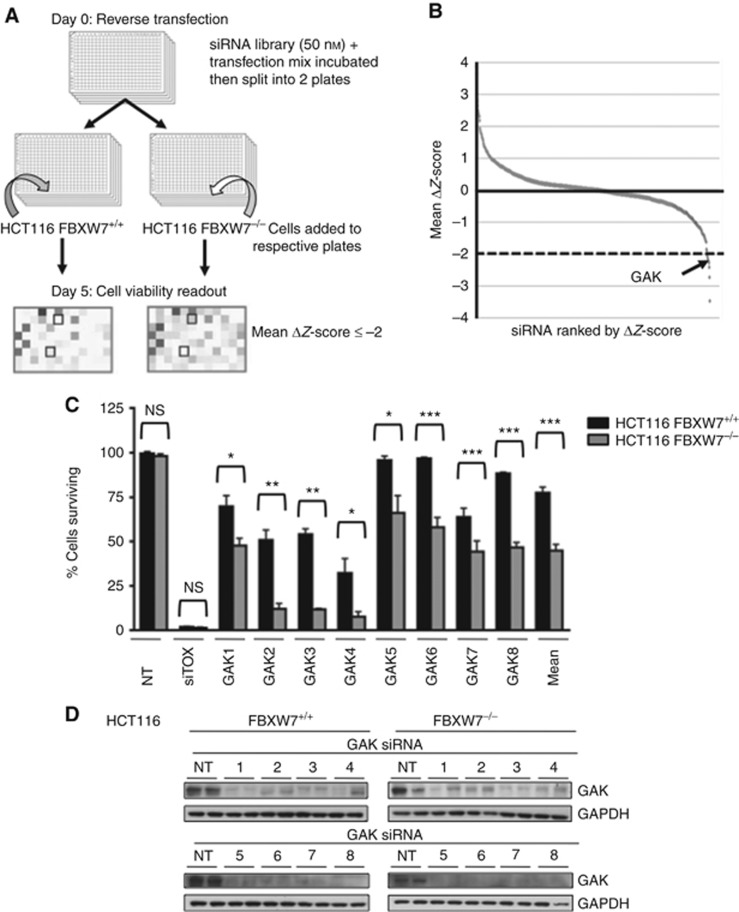
**The RNAi synthetic lethal screen.** (**A**) Schema of siRNA kinome screen. (**B**) Ranked mean Δ*Z*-scores, kinase hit threshold ⩽−2 (*n*=2). (**C**) Quantification of percentage survival of FBXW7^+/+^ (black) and ^−/−^ (grey) HCT116 cells 5 days post transfection with 8 individual GAK siRNAs from the deconvoluted siGENOME (1–4) and ON-TARGET plus (5–8) SMARTpools (*n*=3); mean±s.e.m. Paired *T*-test, *P<0.05, **P<0.01, ***P<0.001; NS, not significant. (**D**) Immunoblot showing GAK and GAPDH levels in FBXW7^+/+^ and ^−/−^ cells 72 h following GAK RNAi.

In this study, we used a FBXW7-deficient HCT116 cell line in order to identify potential genes that cause selective killing in FBXW7-deficient cells compared with the wild-type cells (Jallepalli *et al*, 2001; Rajagopalan *et al*, 2004). The FBXW7^−/−^ cells contain biallelic deletion of the FBXW7 F-box domain resulting in a nonfunctional protein (if expressed) unable to bind or degrade its substrates. High-throughput screening was undertaken by reverse transfection of 720 SMARTpool siRNAs targeting the human protein kinase library (Dharmacon) in 96-well format (Figure 1A), alongside NT and a positive death (siTOX) controls, with the screen readout being cell viability on day 5 (Drosopoulos *et al*, 2014). The screen, conducted in duplicate, was highly reproducible (*r*^2^=0.89 and 0.85) and robust (mean *Z*′ factors of 0.74 and 0.73) for FBXW7 wild-type and FBXW7^−/−^ HCT116 cells, respectively (Supplementary Figure 1). The ranked mean Δ*Z*-scores of the two runs are shown in Figure 1B, and a statistically significant threshold of Δ*Z*-score ⩽−2 was used to select hits for further validation. A secondary RNAi screen was undertaken to validate hits through the concept of redundancy and to exclude target effects of the siRNAs used for the screen; genes would only be considered hits when at least two out of the four oligonucleotides causes selective cell death in FBXW7^−/−^ cells as compared with the wild-type cells. The top 22 primary screen hit SMARTpools were deconvoluted into their 4 individual siRNAs and tested in triplicate under the primary screen conditions, with 7 potential hits validating (Supplementary Table 1). The candidate gene, GAK, was validated as the lead hit, with all 8 individual siRNAs (deconvoluted from two SMARTpools) producing a statistically significant increase in cell death in the FBXW7^−/−^ compared with parental HCT116 cells (Figure 1C: mean 44.88%±3.15 s.e.m. *vs* 77.50%±3.52, respectively). Furthermore, cell death correlated with GAK gene silencing, with robust GAK protein knockdown with all 8 siRNAs (Figure 1D). GAK, or Auxilin 2, is a ubiquitously expressed cytosolic kinase involved in mitosis and receptor-mediated endocytosis (Kimura *et al*, 1997). The role of GAK in mitosis has been presented mainly in mitotic spindle assembly and chromosomal alignment (Shimizu *et al*, 2009; Tanenbaum *et al*, 2010). The GAK functions in receptor-mediated endocytosis, through the formation of clathrin-coated vesicles, involving the epidermal and insulin growth factor receptors (Zhang *et al*, 2004; Susa *et al*, 2010).

**Figure 2:**
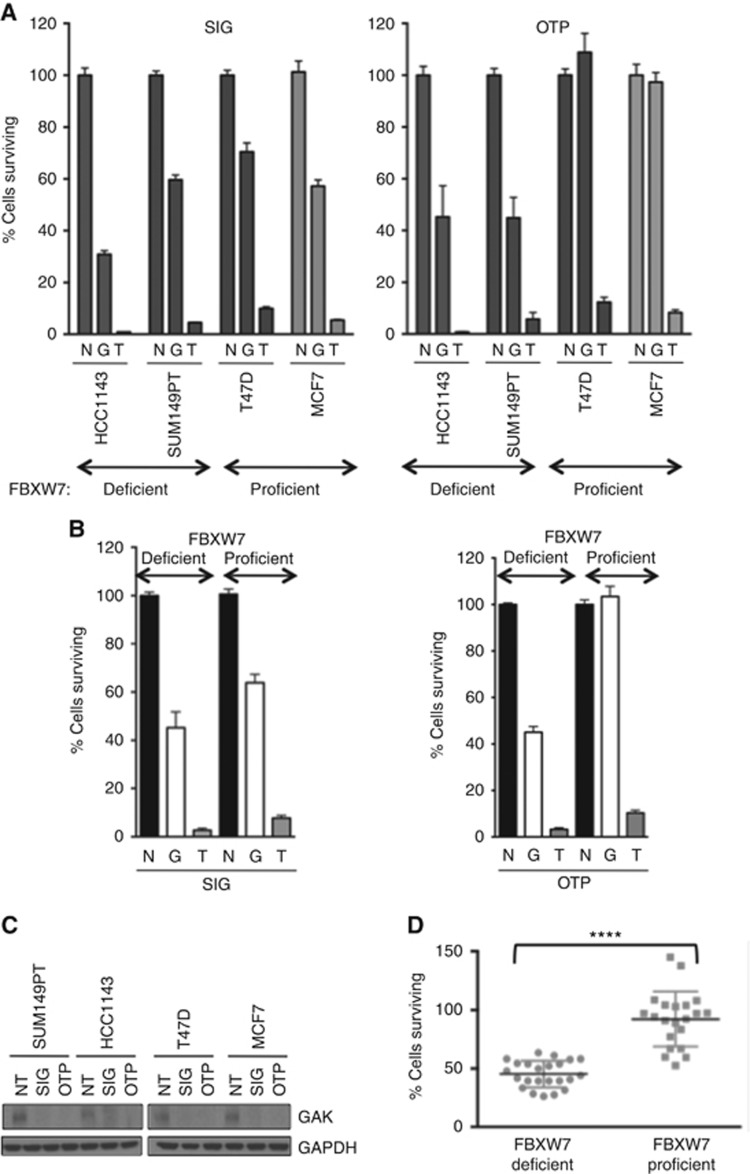
**Validation of FBXW7-GAK synthetic lethality in breast cells.** (**A**) Quantification of cell survival 5 days after transfection with GAK (G) siGENOME (SIG), left panel, and ON-TARGET plus (OTP) siRNAs, right panel, relative to nontargeting (N) and siTOX (T) controls (*n*=3). (**B**) Median cell survival of the combined breast FBXW7-proficient *vs* -deficient cells with N, T and G SIG and OTP siRNA. (**C**) Immunoblot of GAK and GAPDH 72 h post GAK SMARTpool RNAi. (**D**) Combined analysis of breast cell survival by FBXW7 status using paired *T*-test, ****P<0.0001.

**Figure 3:**
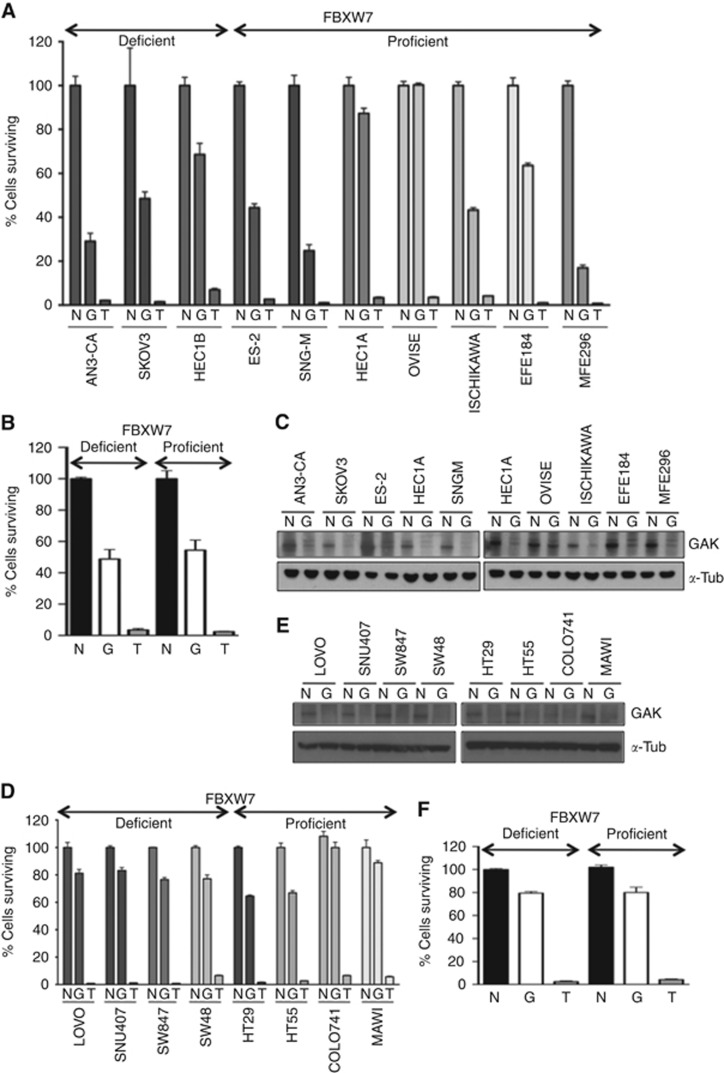
**The FBXW7-GAK synthetic lethal relationship is not maintained in FBXW7-deficient gynaecological or colorectal cancer cell lines.** (**A** and **D**) Percentage cell survival 5 days post transfection with GAK siGENOME (G) siRNA compared with nontargeting (N) and siTOX (T) controls in gynaecological and colorectal cells, respectively (*n*=3). (**B** and **F**) Combined analysis of gynaecological (**B**) and colorectal (**F**) cell survival by FBXW7 status, NS by paired *T*-test. (**C** and **E**) Immunoblot of GAK and *α*–-tubulin (*α*-tub) 72 h post GAK RNAi in gynaecological (**C**) and colorectal (**F**) cells.

To further test whether GAK RNAi caused a reduction in proliferation of FBXW7-deficient cells, GAK RNAi was used in 22 cell lines (9 FBXW7-deficient and 13 proficient wild-type cell lines): 4 breast, 10 gynaecological and 8 colorectal (Supplementary Table 2). Given the small number of FBXW7-mutant cell lines known and available, it was not possible to select cells of similar histological or biological subtypes. Rigorous optimisation was undertaken to identify the optimal conditions to ensure the least toxicity and most efficient transfections for each cell line (Figures 2 and 3).

The breast FBXW7-deficient (HCC1143, SUM149PT) and FBXW7-proficient (T47D, MCF7) cells demonstrated a clear relationship between FBXW7 loss of function and sensitivity to GAK knockdown, using two separate GAK SMARTpools, siGENOME and OTP (Figure 2A). Combined analysis of the two FBXW7-deficient cell lines showed a significantly reduced median cell viability of 45.3 and 45.1%, compared with 63.8 and 98.6% for the two wild-type comparators, for the respective SMARTpools (Figure 2B). This accorded with robust GAK protein knockdown (Figure 2C). To identify statistical significance the combined analysis of all the aforementioned breast cancer cell lines using both SMARTpools was undertaken. Each data point represents cell survival of the FBXW7-deficient HCC1143 and SUM149PT cells compared with the proficient T47D and MCF7 cells with the siGENOME and OTP SMARTpools from triplicate experiments. This confirmed a highly statistically significant difference for the FBXW7-deficient cells with median survival of 43.8 *vs* 94.2%, *P*<0.0001 by paired *T*-test (Figure 2D).

FBXW7-deficient gynaecological cell lines were not more sensitive to GAK RNAi compared with the FBXW7 wild-type cells (Figure 3A and B). Median cell survival was 46% *vs* 53% in FBXW7-deficient and -proficient cells prospectively (Figure 3B) despite adequate protein knockdown (Figure 3C). Furthermore, the entire colon cancer cell panel was relatively insensitive to GAK inhibition (Figure 3D) despite good protein knockdown (Figure 3E). The cells showed similar low sensitivity to GAK RNAi whether they were FBXW7 proficient or deficient, with median cell viabilities of 84% and 81%, respectively (Figure 3F). Hence, GAK depletion is not synthetically lethal to FBXW7 loss in colorectal or gynaecological cell lines. The small number of FBXW7-mutant cell lines available hindered investigation in a larger cell line panel.

### Dual GAK-FBXW7 inhibition induces apoptosis after 48 h

**Figure 4:**
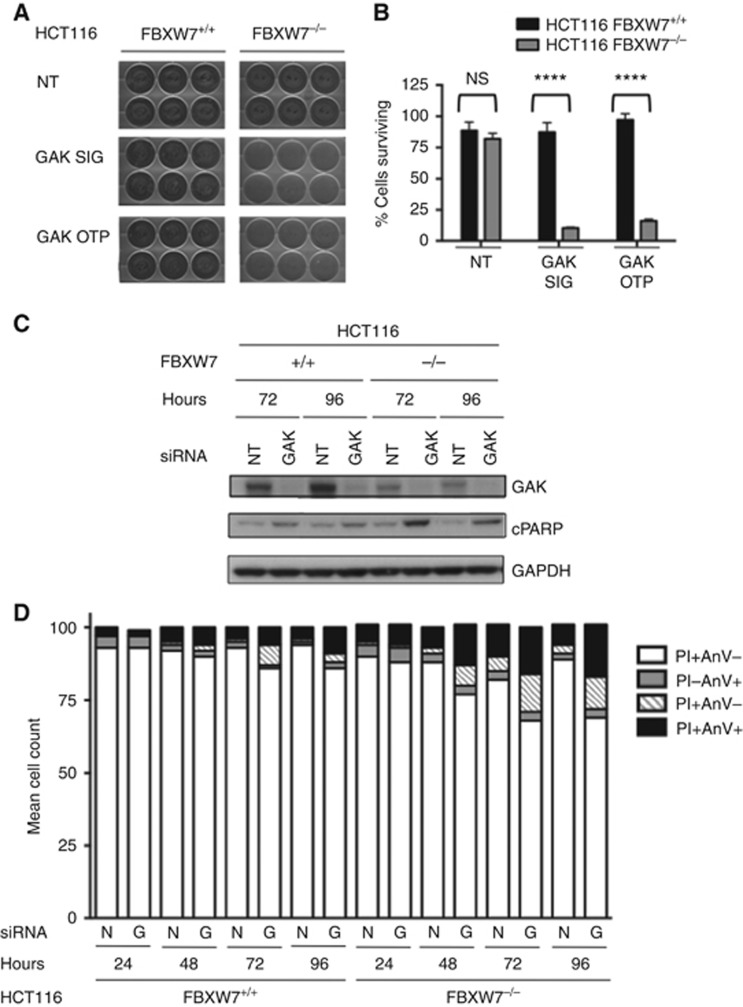
**Dual GAK-FBXW7 inhibition induces apoptosis in HCT116 FBXW7-deficient cells.** (**A** and **B**) Long-term proliferation assay of FBXW7^+/+^ and ^−/−^ HCT116 cells 14 days following RNAi with GAK siGENOME (SIG) and ON-TARGET plus (OTP) compared with nontargeting (NT) siRNA. (**A**) Images of fixed and stained cells. (**B**) Quantification of sulforhodamine B assay by paired *T*-test, ****P<0.0001. (**C**) Immunoblot of GAK, cleaved PARP (cPARP) and GAPDH 72 and 96 h post GAK and NT RNAi. (**D**) Annexin V assay of FBXW7^+/+^ and ^−/−^ HCT116 cells at 24–96 h following GAK siGENOME SMARTpool (G) and nontargeting (N) siRNA. Cells are deemed apoptotic if they stain for propidium iodide (PI) and/or Annexin V (AnV) or viable if they are PI and AnV positive (+) or negative (−).

To analyse the mode of death in the FBXW7^−/−^ HCT116 cells, a long-term cell proliferation assay was performed for 14 days using two SMARTpools targeting GAK (Figure 4A and B). This confirmed that GAK RNAi was significantly more toxic to FBXW7^−/−^ compared with the wild-type cells (Figure 4A), reducing cell viability to ∼10% with the siGENOME and 16% with the OTP pools, compared with 87 and 97% viability for FBXW7^+/+^ cells, respectively (Figure 4B). This is likely to indicate incremental FBXW7^−/−^ killing over a prolonged time period, as the percentage cell death is higher at 14 as compared with 5 days despite the fact the RNAi-mediated gene silencing would have ended.

The PARP cleavage was used to determine whether the cells were undergoing apoptosis at 72 and 96 h following GAK RNAi (Figure 4C). In association with robust GAK protein knockdown following RNAi, there was a clear increase in cleaved PARP levels at both 72 and 96 h in the F-box deficient but not wild-type cells, suggesting apoptosis had already commenced at these times. This confirmed GAK-mediated cell death by apoptosis preferentially in the FBXW7^−/−^ HCT116 cells after 72 h as compared with the wild-type cells. Interestingly, it was noticed that GAK levels are lower in the FBXW7^−/−^ cells, although the reason for this is unclear. An Annexin V assay was undertaken to quantify the degree of apoptosis in the FBXW7 isogenic cells at 24–96 h following siRNA transfection with GAK siGENOME SMARTpool compared with the NT control (Figure 4D). Similar results were obtained for the FBXW7-proficient and -deficient cells with the control and GAK siRNA at 24 h; mean viable cells were 93–97% *vs* apoptotic cells of 2–8%. From 48 h onwards, the GAK RNAi consistently induced double the number of apoptotic cells in FBXW7^−/−^ compared with the wild-type cells; 21% *vs* 8% at 48 h and 29% *vs* 12% at 96 h, respectively, thus, confirming a preferential increase in apoptosis in the FBXW7^−/−^ cell line in response to GAK RNAi compared with the wild-type controls from 48 h incrementing to involve a fifth of cells by 72 h (Figure 4D).

### Dual GAK-FBXW7 inhibition causes cell cycle disruption and perturbs mitosis

**Figure 5:**
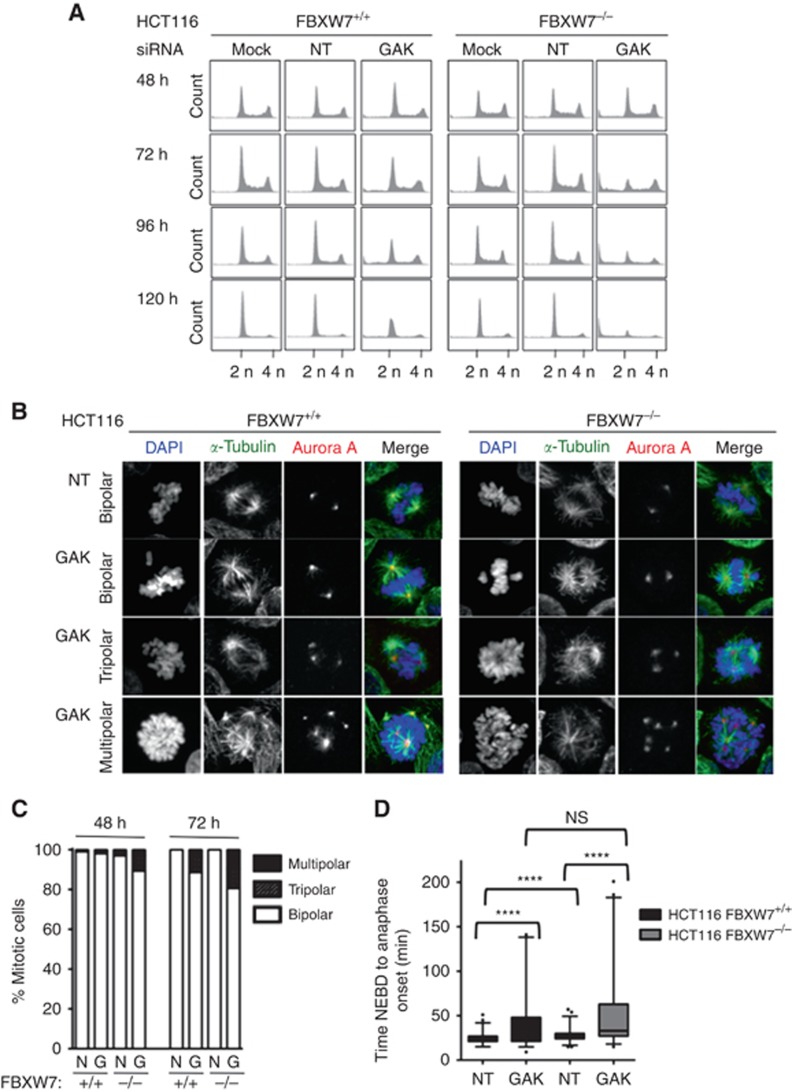
**The GAK silencing in FBXW7-deficient cells perturbs mitosis.** (**A**) DNA content of asynchronous FBXW7^+/+^ and ^−/−^ HCT116 cells at indicated time points following transfection with mock, nontargeting (NT) and GAK siRNA. Propidium iodide (PI) staining was analysed by flow cytometry. (**B**) Immunofluorescence of PEM fixed FBXW7^+/+^ and ^−/−^ HCT116 cells stained with DAPI (blue), *α*-tubulin (green) and Aurora A (red) 72 h following transfection with NT and GAK siRNA. (**C**) Quantification of multipolar mitoses identified by immunofluorescence at 48 and 72 h post GAK siGENOME RNAi. (**D**) Mitotic duration at 48 h following GAK and NT siRNA, measured from time of nuclear envelope breakdown (NEBD) to anaphase onset in FBXW7^+/+^ (black) and ^−/−^ (grey) HCT116 cells stably expressing histone H2B-mCherry, *n*=100. Paired Student’s *T*-tests, ****P<0.0001.

The GAK RNAi has previously been reported to cause a mitotic arrest in HeLa cells (Shimizu *et al*, 2009), and we assessed the global cell cycle effect of GAK RNAi on the FBXW7 isogenic cells using flow cytometry. The HCT116 cells were treated with mock, NT and GAK siGENOME SMARTpool siRNA, and then the DNA content analysed by PI staining 48–120 h later (Figure 5A). At 48 h, the cell cycle profiles for both cell lines under all conditions were similar, with a clear large 2n DNA peak (G1 cells) and smaller 4n DNA peak (G2/M). However, from 72 h onwards, a clear difference became evident between the FBXW7-deficient and -proficient cells when treated with the GAK siRNA. For the wild-type cells, at 72 h there is a small decrease in the 2n population; however, in the FBXW7-null cells, this phenotype is much more dramatic, with the 2n peak being only as large as the 4n peak, with a concomitant increase in the sub-2n population (apoptotic cells). However, as the 4n peak remains the same size, this argues against a significant cell cycle arrest in G2 or mitosis. Furthermore, this phenotype gets progressively more intense over time, with a complete loss of the cell cycle by 120 h, although the wild-type cells appear to be less severely affected by the GAK RNAi.

Multipolar spindle formation in HeLa cells has also been reported following GAK RNAi (Shimizu *et al*, 2009). To ascertain whether this holds true in HCT116 cells and to determine whether this phenotype is exacerbated in FBXW7^−/−^ cells, immunofluorescence microscopy was performed at 48 and 72 h following RNAi with NT and GAK SMARTpool RNAi in the HCT116 isogenic pair (Figure 5B and C). Cells were fixed and stained with antibodies against *α*-tubulin and Aurora A to visualise the mitotic spindles and centrosomes (Figure 5B), respectively, and the number of multipolar cells quantified (Figure 5C). In both cells transfected with the NT control, the majority of mitotic cells contained bipolar spindles, with only a very small number of multipolar mitoses being evident. However, following GAK RNAi, there was a clear induction in multipolar mitoses in both cell lines. The FBXW7^−/−^ cells demonstrated a two-fold increase in combined tri- and multipolar spindles compared with wild-type cells with 11% *vs* 2%, and 19% *vs* 11%, at 48 and 72 h, respectively (Figure 5C). Importantly, not only was this phenotype more prominent in the FBXW7 null cells, but it was more severe, with up to 6 spindle poles being identified in some cells, whereas generally only tripolar formations were seen in the wild-type cells (Figure 5B). This suggests that the loss of GAK and FBXW7 may synergise in producing multipolar spindles.

The GAK RNAi-induced mitotic arrest has been documented in HeLa and U2OS cell lines (Shimizu *et al*, 2009). In addition to the cell cycle disruption identified predominantly in F-box-deficient cells, we determined the effect of GAK silencing on mitotic duration using time-lapse microscopy. The FBXW7^+/+^ and ^−/−^ HCT116 cells were made to stably express histone H2B-mCherry, allowing the visualisation of the DNA. Time-lapse microscopy was performed 48 h following transfection with NT or GAK SMARTpool siRNA (Figure 5D). Mitotic duration was calculated for 100 cells for each siRNA. Although small, we observed a statistically significant difference in the median mitotic duration for the NT controls between the FBXW7-proficient and -deficient cells: 21 *vs* 27 min, respectively (*n*=100, *P*<0.0001), perhaps suggesting a tendency for the FBXW7-null cells to have more problems aligning their chromosomes, as previously reported (Rajagopalan *et al*, 2004; Bailey *et al*, 2015). For the cells treated with GAK RNAi, the median mitotic interval was increased to 30 min (95% CI 33.67–46.73) *vs* 33 min (43.03–57.59), respectively. Although this was not significantly different between the isogenic cells (*n*=100, *P*=0.23), it was constantly longer than the NT controls. This indicates that GAK does appear to play a role in the timing of mitosis in HCT116 cells; however, it appears to be relatively minor and independent of FBXW7 function. Such a small increase may also explain why a difference was not apparent by flow cytometry.

### Synthetic lethal mechanism between FBXW7 and GAK is clathrin independent

**Figure 6:**
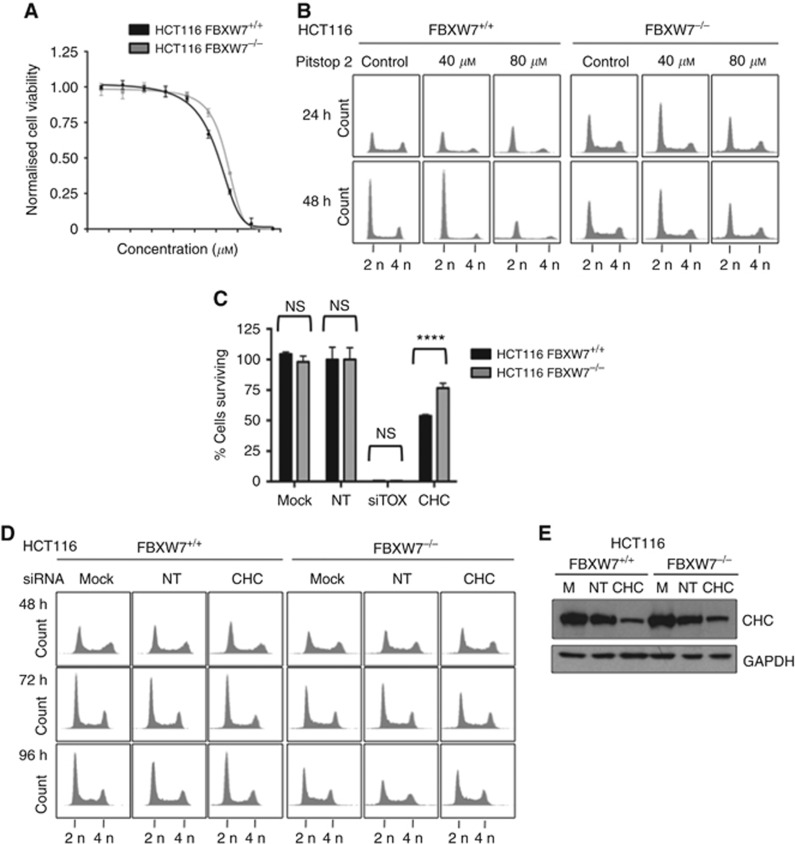
**Clathrin inhibition through RNAi and Pitstop 2 does not mirror phenotype observed with GAK inhibition in isogenic HCT116 cells.** (**A**) Pitstop 2 growth inhibition curves of % survival fraction of FBXW7^+/+^ (black) and ^−/−^ (grey) HCT116 at day 5. Growth inhibitory concentrations (GI_50_) were 30.51 *μ*M (95% CI 25.9–35) and 43.81 *μ*M (95% CI 41.7–45.9), respectively; not significant by *T*-test (*P*=0.3). (**B**) DNA content of asynchronous FBXW7^+/+^ and ^−/−^ HCT116 cells at 24 and 48 h post treatment with Pitstop 2 at the indicated concentrations. (**C**) Quantification of cell survival following mock, nontargeting (NT) and clathrin heavy chain (CHC) RNAi, compared by paired *T*-tests, ****P<0.0001. (**D**) Propidium iodide staining (DNA content) for asynchronous cells 48–96 h following transfection with mock, NT and CHC siRNA. (**E**) Immunoblot of CHC and GAPDH for HCT116 FBXW7^+/+^ and ^−/−^ cells 72 h post RNAi.

Pitstop 2, a cell-permeable clathrin inhibitor (Dutta *et al*, 2012), and siRNA targeting the clathrin heavy chain (CHC) were utilised to evaluate whether the synthetic lethal mechanism with FBXW7 was related to the clathrin function of GAK (Figure 6). In viability assays similar killing curves were apparent in the FBXW7 isogenic HCT116 cells following 5-day exposure to Pitstop 2 (Figure 6A). Actually, the FBXW7^−/−^ cells seemed to be slightly more insensitive to clathrin inhibition. Cell cycle profiling was undertaken at 24–48 h following Pitstop 2 treatment at 40 and 80 *μ*M (Figure 6B). A similar profile was seen for the controls at both concentrations and time points, with Pitstop 2 appearing to have no effect on the cell cycle profiles, with the exception of 80 *μ*M at 48 h in the wild-type cells. These cell cycle profiles are markedly different to the same cell lines in response to GAK RNAi (Figure 5A). This hypothesis was tested further using RNAi against clathrin (Figure 6C–E). Once again the FBXW7-deficient HCT116 cells were less sensitive to clathrin siRNA than the wild-type cells when using a proliferation assay, with statistically significantly different mean cell survival of 77% compared with 54% in the proficient comparators (Figure 6C); this correlates with the Pitstop 2 drug response. Secondly, there was no obvious alteration in the cell cycle profiles of either the FBXW7^+/+^ or ^−/−^ cells at 48–96 h post clathrin siRNA transfection (Figure 6D) despite adequate clathrin knockdown (Figure 6E). All these data together suggest that the toxicity associated with GAK knockdown is not mediated through clathrin binding in these FBXW7 isogenic cells.

## Discussion

A robust siRNA kinome screen identified GAK as a synthetic lethal target with FBXW7 loss. The use of SMARTpools maximised knockdown efficiency while minimising the individual siRNA dose. The GAK validated strongly with 8 out of 8 different siRNAs inducing statistically significant preferential death in HCT116 FBXW7^−/−^ with associated efficient gene silencing of the GAK protein. This markedly reduces the risk of these findings being due to off-target effects or activation of the interferon response (Sharma and Rao, 2009; Kaelin, 2012).

Silencing of GAK, by two different SMARTpools, in breast basal FBXW7-deficient cells caused statistically significant increased cell death of 19% and 54% relative to the wild-type comparators. The FBXW7-GAK synthetic lethal relationship was not confirmed in the colorectal or gynaecological cell lines. This may be due to the fact that different molecular backgrounds (Supplementary Table 2) define sensitivity, such as is apparent clinically with the contrasting responses seen with single agent BRAF inhibitors in melanoma and colorectal cancers (Herr and Brummer, 2015). Wider exploration in larger cell line panels is not possible given the small numbers of FBXW7-mutant cells available.

We have demonstrated that GAK inhibition in FBXW7^−/−^ HCT116 causes cell death that increases over time, detected by a variety of assays. From 48 h, the cell cycle is disrupted, followed by the induction of apoptosis at 72 h. By day 5, cell death was at 60–80%, which increased to 85–90% by day 14. The prolonged nature of these effects suggests accumulation of cellular defects is necessary before death ensues.

The work conducted focussed on the role of GAK in mitotic spindle assembly and chromosomal alignment (Shimizu *et al*, 2009; Tanenbaum *et al*, 2010). We have demonstrated that siRNA-mediated GAK silencing causes marked cell cycle disruption resulting in a raised sub-G1 alongside decreased G1 and G2/M cell cycle phases. Published work in HeLa cells has shown GAK RNAi induces mitotic arrest and increased G2/M peak (Shimizu *et al*, 2009). This discrepancy may simply reflect the different cell lines used. In addition, the true phenotype of cellular GAK depletion has not reached a consensus, with conflicting data reported from different cell lines and groups (Lee *et al*, 2008). Contrary to GAK RNAi inducing mitotic arrest in HeLa cells (Shimizu *et al*, 2009), there was only a minor mitotic delay of 3 min in both the FBXW7^+/+^ and ^−/−^ HCT116 cells, but this was neither significant nor specific to loss of F-box functionality, suggesting that GAK is involved, but not essential for, mitotic progression concurring with previous reports (Shimizu *et al*, 2009). The differences between our and published data may purely represent different cell line responses to GAK knockdown.

The most marked phenotype of GAK RNAi-induced multipolar mitoses that appeared to be augmented in the context of FBXW7 loss represents a notable outcome and mirrored data in the HeLa cell lines (Shimizu *et al*, 2009). Dual FBXW7-GAK inhibition increases multipolar mitoses to ∼20% at 72 h. This doubling, compared with the wild-type controls, could account for the increased cell death potentially through chromosomal missegregation resulting in aneuploidy (Duensing and Munger, 2001). This mechanism may account for the selective toxicity of GAK siRNA in FBXW7-deficient cells known to possess chromosomal instability, mainly documented by cyclin E overexpression (Spruck *et al*, 1999; Rajagopalan *et al*, 2004; Loeb *et al*, 2005). The accumulation of mitotic defects through GAK inhibition could present a therapeutic strategy by inducing replicative stress, in a selective manner, targeting the inherent mitotically unstable FBXW7^−/−^ cells (Dobbelstein and Sorensen, 2015).

We have identified a potential novel drug candidate for FBXW7-deficient tumours. The phenotype of mutual GAK-FBXW7 loss is unlikely to have resulted from clathrin or receptor-mediated endocytosis. The severe cell cycle disruption with multipolar defects means that it is more likely to be a mitotic defect, although elucidating the exact mechanism of cell death has proven difficult. The GAK-FBXW7-deficient phenotype appears different to GAK inhibition in other cells (Shimizu *et al*, 2009; Tanenbaum *et al*, 2010) and not related to loss of the spindle assembly checkpoint in HCT116 cells (Bailey *et al*, 2015). Currently, there are no commercially available selective GAK inhibitors to allow the use of kinase inhibitors to simulate siRNA effects. Data have recently been published on the development of a GAK-specific inhibitor, although the potency is relatively low with the half-maximal concentration (EC_50_) values in the 2–3 *μ*M range (Kovackova *et al*, 2015); we were unable to access this for confirmation experiments at this juncture. Further studies are needed warranted including expansion of cell line work by blocking FBXW7 activity in wild-type cells as well as to ascertaining whether GAK inhibition is kinase dependent.

A multitude of cancers would be good clinical candidates for specific GAK inhibitors including cholangiocarcinoma, as epithelial bile duct cancers are one of the highest mutated FBXW7 tumours, have poor prognosis and a paucity of therapeutic agents (Meza-Junco *et al*, 2010). Our data also support the potential use of GAK inhibitors in triple-negative FBXW7-deficient tumours. During the development of any novel inhibitor, it is imperative to establish a robust predictive biomarker that would be key to selecting preferential candidate compounds.
