# Supplementary figures and images for: RNAi screen reveals synthetic lethality between cyclin G-associated kinase and FBXW7 by inducing aberrant mitoses
Source: Br J Cancer. 2017 Aug 22;117(7):954–64. doi: 10.1038/bjc.2017.277 (PMC5625678; doi:10.1038/bjc.2017.277)

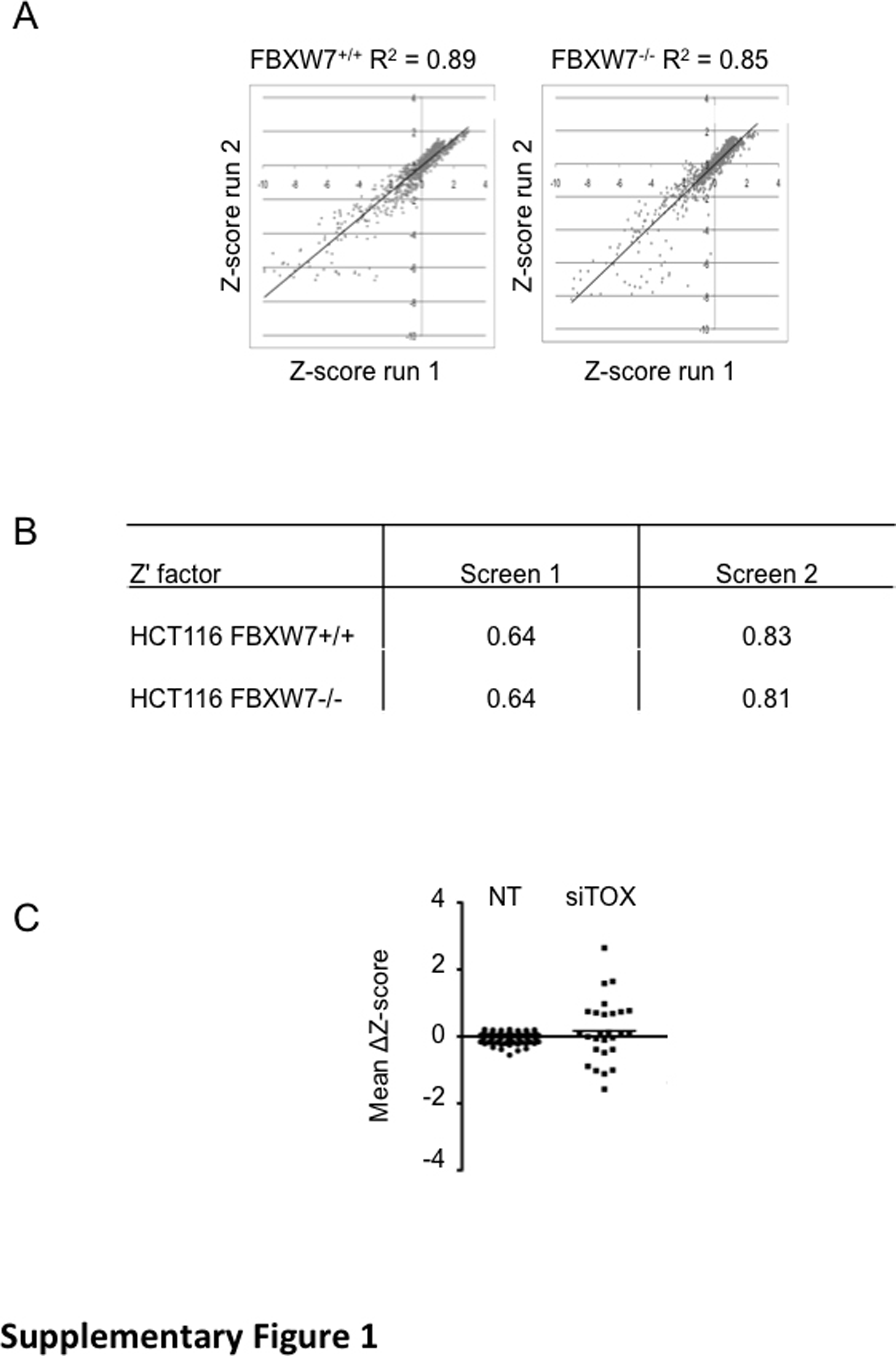

Supplement: Supplementary Figure 1 [file bjc2017277x1.tif]
