# Supplementary material for: RNAi screen reveals synthetic lethality between cyclin G-associated kinase and FBXW7 by inducing aberrant mitoses
Source: Br J Cancer. 2017 Aug 22;117(7):954–64. doi: 10.1038/bjc.2017.277 (PMC5625678; doi:10.1038/bjc.2017.277)
Supplement: Supplementary Table 1 [file bjc2017277x2.docx]

**Supplementary Table 1: Top hits from the secondary screen using the siGENOME deconvoluted siRNA**

| **siRNA** | **Full Name** | **% Cell viability HCT116** | | **p value** |
| --- | --- | --- | --- | --- |
|  |  | **FBXW7^+/+^** | **FBXW7^-/-^** |  |
| NT | Non targeting control | 100.0 | 100.0 | 0.50 |
| siTOX | siTOX control | 2.0 | 2.2 | 0.40 |
| GAK | Cyclin G associated kinase | 69.8 | 47.7 | 0.02 |
| GAK |  | 50.8 | 12.1 | 0.00 |
| GAK |  | 54.0 | 11.8 | 0.00 |
| GAK |  | 32.3 | 7.8 | 0.02 |
| BUB1B | Budding uninhibited by benzimidazoles 1 | 50.6 | 36.5 | 0.04 |
| BUB1B |  | 63.1 | 35.4 | 0.01 |
| BUB1B |  | 50.3 | 25.4 | 0.01 |
| BUB1B |  | 19.3 | 17.2 | 0.31 |
| FGFR1 | Fibroblast growth factor receptor 1 | 49.2 | 25.1 | 0.05 |
| FGFR1 |  | 64.5 | 45.5 | 0.03 |
| FGFR1 |  | 63.9 | 48.5 | 0.07 |
| FGFR1 |  | 78.1 | 71.5 | 0.12 |
| BCKDK | Branched chain ketoacid dehydrogenase | 3.1 | 2.4 | 0.00 |
| BCKDK |  | 92.0 | 78.9 | 0.02 |
| BCKDK |  | 107.6 | 94.1 | 0.10 |
| BCKDK |  | 40.9 | 40.7 | 0.47 |
| RET | RET proto-oncogene | 78.3 | 60.6 | 0.01 |
| RET |  | 24.9 | 6.9 | 0.00 |
| RET |  | 14.4 | 12.9 | 0.12 |
| RET |  | 86.1 | 86.3 | 0.46 |
| FN3KRP | Fructosamine 3 kinase related protein | 66.7 | 50.8 | 0.02 |
| FN3KRP |  | 34.1 | 21.7 | 0.05 |
| FN3KRP |  | 34.2 | 41.5 | 0.11 |
| FN3KRP |  | 30.9 | 40.9 | 0.00 |
| TRIB2 | Tribbles homolog 2 | 7.6 | 4.6 | 0.00 |
| TRIB2 |  | 24.8 | 13.9 | 0.01 |
| TRIB2 |  | 101.7 | 100.4 | 0.41 |
| TRIB2 |  | 102.1 | 104.7 | 0.26 |
