# Supplementary material for: RNAi screen reveals synthetic lethality between cyclin G-associated kinase and FBXW7 by inducing aberrant mitoses
Source: Br J Cancer. 2017 Aug 22;117(7):954–64. doi: 10.1038/bjc.2017.277 (PMC5625678; doi:10.1038/bjc.2017.277)
Supplement: Supplementary Table 2 [file bjc2017277x3.docx]

**Supplementary Table 2: Overview of breast, gynaecological and colon cancer cell lines used to validate FBXW7-GAK synthetic lethal partnership**

| **Cell line** | **Tumour type** | **FBXW7 alteration** | **FBXW7 phenotype** | **Other mutations** | | | | |
| --- | --- | --- | --- | --- | --- | --- | --- | --- |
|  |  |  |  | PIK3CA | P53 | PTEN | RAS/RAF | Notable Others |
| **BREAST** | | | | | | | | |
| SUM149PT | Breast: basal B IDC ER- PR- | F549fs*6 | Homozygous nonsense mutation causing truncation of WD40 |  |  |  |  |  |
| HCC1143 | Breast: basal A IDC ER- PR- | Wildtype | Mono-allelic deletion of FBXW7 gene |  |  |  |  |  |
| T47D | Breast: luminal A IDC ER+ PR+ | Wildtype |  | + | + |  |  |  |
| MCF7 | Breast: luminal A IDC ER+ PR+ | Wildtype |  | + |  |  |  |  |
| **GYNAECOLOGICAL** | | | | | | | | |
| AN3-CA | Endometrial: adenocarcinoma | R441W | Heterozygous missense WD40 |  | + |  |  | MET |
| SK-O-V3 | Ovarian: adenocarcinoma | R505L | Heterozygous missense WD40 of conserved arginine | + |  |  |  | NF1 |
| HEC1B | Endometrial: adenocarcinoma | R367* | Nonsense upstream of WD40 |  |  |  | +  KRAS  HRAS |  |
| ES-2 | Ovarian: clear cell carcinoma | Wildtype |  |  |  |  | +  BRAF |  |
| MFE-296 | Endometrial: adenocarcinoma | Wildtype |  | + | + | + |  |  |
| ISHIKAWA | Endometrial: adenocarcinoma | Wildtype |  |  | + | + |  |  |
| EFE184 | Endometrial:  carcinoma | Wildtype |  |  |  |  |  |  |
| OVISE | Ovarian: adenocarcinoma | Wildtype |  |  |  |  |  |  |
| SNG-M | Endometrial: adenocarcinoma | Wildtype |  | + |  | + |  |  |
| HEC1A | Endometrial: adenocarcinoma | Wildtype |  | + |  |  |  | NF1 |
| **COLON** | | | | | | | | |
| LoVo | Colon:  adenocarcinoma | R505L | Heterozygous missense WD40 of conserved arginine |  |  |  | + | NF1 |
| SNU407 | Colon:  adenocarcinoma | R465C | Heterozygous missense WD40 of conserved arginine | + |  | + | +  BRAF |  |
| SW837 | Rectal:  adenocarcinoma | L403fs*34 | Heterozygous Frameshift in WD40 |  |  |  | +  KRAS |  |
| SW48 | Colon:  adenocarcinoma | S668fs*39 | Heterozygous Frameshift in WD40 | + |  | + | +  HRAS | MSH6 |
| HT55 | Colon:  adenocarcinoma | T8S | Homozygous missense |  |  |  | +  BRAF | NF1 |
| HT-29 | Colon:  carcinoma | Wildtype |  | + |  |  | +  BRAF |  |
| COLO-741 | Colon:  carcinoma | Wildtype |  |  |  |  |  | MLH1 |
| MAWI | Colon:  carcinoma | Wildtype |  |  |  |  | +  BRAF |  |
