# Supplementary material for: RNAi screen reveals synthetic lethality between cyclin G-associated kinase and FBXW7 by inducing aberrant mitoses
Source: Br J Cancer. 2017 Aug 22;117(7):954–64. doi: 10.1038/bjc.2017.277 (PMC5625678; doi:10.1038/bjc.2017.277)
Supplement: Supplementary Figure Legend [file bjc2017277x4.docx]

**RNAi screen reveals synthetic lethality between Cyclin G-associated kinase and FBXW7 by inducing aberrant mitoses**

**Authors:** Saoirse O. Dolly^1,2^, Mark D. Gurden^3^, Konstantinos Drosopoulos^3^, Paul Clarke^1^, Johann de Bono^1,2^, Stan Kaye^1,2^, Paul Workman^1^ and Spiros Linardopoulos^1,3^.

**Affiliations:**

^1^Cancer Research UK Cancer Therapeutics Unit, Division of Cancer Therapeutics, The Institute of Cancer Research, London, UK; ^2^Royal Marsden Hospital, London, UK; ^3^Breast Cancer Now, Division of Breast Cancer Research, The Institute of Cancer Research, London, London, UK.

**Supplementary Figure 1: Kinome siRNA screen metrics. A.** Correlation curves of Z-scores of the biological duplicates performed for individual wells for each cell line. The linear regression coefficient (R^2^) for run 1 versus run 2 FBXW7^+/+^ and ^-/-^ HCT116 cells was 0.89 and 0.85, respectively. **B.** Mean Z’ factor for the isogenic cells biological duplicate replicates. **C.** Scatter plots of mean ΔZ-scores for the non-targeting (NT) and siTOX siRNA controls.
